# An Efficient Combination among sMRI, CSF, Cognitive Score, and *APOE ε*4 Biomarkers for Classification of AD and MCI Using Extreme Learning Machine

**DOI:** 10.1155/2020/8015156

**Published:** 2020-06-04

**Authors:** Uttam Khatri, Goo-Rak Kwon

**Affiliations:** Department. of Information and Communication Engineering, Chosun University, 309 Pilmun-Daero, Dong-Gu, Gwangju 61452, Republic of Korea

## Abstract

Alzheimer's disease (AD) is the most common cause of dementia and a progressive neurodegenerative condition, characterized by a decline in cognitive function. Symptoms usually appear gradually and worsen over time, becoming severe enough to interfere with individual daily tasks. Thus, the accurate diagnosis of both AD and the prodromal stage (i.e., mild cognitive impairment (MCI)) is crucial for timely treatment. As AD is inherently dynamic, the relationship between AD indicators is unclear and varies over time. To address this issue, we first aimed at investigating differences in atrophic patterns between individuals with AD and MCI and healthy controls (HCs). Then we utilized multiple biomarkers, along with filter- and wrapper-based feature selection and an extreme learning machine- (ELM-) based approach, with 10-fold cross-validation for classification. Increasing efforts are focusing on the use of multiple biomarkers, which can be useful for the diagnosis of AD and MCI. However, optimum combinations have yet to be identified and most multimodal analyses use only volumetric measures obtained from magnetic resonance imaging (MRI). Anatomical structural MRI (sMRI) measures have also so far mostly been used separately. The full possibilities of using anatomical MRI for AD detection have thus yet to be explored. In this study, three measures (cortical thickness, surface area, and gray matter volume), obtained from sMRI through preprocessing for brain atrophy measurements; cerebrospinal fluid (CSF), for quantification of specific proteins; cognitive score, as a measure of cognitive performance; and *APOE ε*4 allele status were utilized. Our results show that a combination of specific biomarkers performs well, with accuracies of 97.31% for classifying AD vs. HC, 91.72% for MCI vs. HC, 87.91% for MCI vs. AD, and 83.38% for MCIs vs. MCIc, respectively, when evaluated using the proposed algorithm. Meanwhile, the areas under the curve (AUC) from the receiver operating characteristic (ROC) curves combining multiple biomarkers provided better classification performance. The proposed features combination and selection algorithm effectively classified AD and MCI, and MCIs vs. MCIc, the most challenging classification task, and therefore could increase the accuracy of AD classification in clinical practice. Furthermore, we compared the performance of the proposed method with SVM classifiers, using a cross-validation method with Alzheimer's Disease Neuroimaging Initiative (ADNI) datasets.

## 1. Introduction

Alzheimer's disease (AD) is a progressive and irreversible neurodegenerative disorder of the central nervous system, characterized by abnormal accumulation of neurofibrillary tangles and amyloid plaques in the brain, affecting the behavior, thinking, and memory of an individual [1]. Alzheimer's disease occurs in late life and is the most common form of dementia, for which there is no cure. An estimated 5.7 million Americans are living with AD in 2018. By 2050, this figure is projected to rise to nearly 14 million [2]. Although some currently available treatments may temporarily decelerate the progression, none have demonstrable effectiveness in treating patients with AD. A promising amount of ongoing research [3–6] is focused on different biomarker-based techniques, in an effort to detect early AD-related changes and characterize prominent atrophy patterns during the prodromal stages, when mild symptoms are the only evidence of the disease. Thus, it is important to develop strategies to enable timely treatment and delay progression during the early stages of AD, before the onset of clinical symptoms. As a result, the concept of mild cognitive impairment (MCI) was introduced. MCI, a transitional stage between healthy (normal) controls (HC) and AD patients, is defined to describe people who have mild symptoms of brain dysfunction but can still perform everyday tasks. Identifying potentially highly sensitive diagnostic biomarkers that change with disease progression may support the physician in making a correct diagnosis. If AD is detected during the early stage of MCI, the number of patients could be reduced by nearly one-third, through rehabilitation exercises and appropriate medication [7]. Patients in the MCI stage have a high risk of progressing to dementia [8–10] and can be categorized as stable MCI (MCIs) or convertible MCI (MCIc) which is also known as progressive MCI (pMCI). Some MCI patients progress to AD within a specific time frame, while others remain stable. Reports have shown that 10–15% of MCI patients progress to AD each year, and 80% of these will have converted to AD after approximately 5-6 years of follow-up [9, 11]. It is crucial to find biomarkers that distinguish patients who have MCI and later converted to AD (MCIc), from those who do not convert to AD, and HC. Thus, early identification of MCI individuals is increasingly clinically important in potentially delaying or preventing the conversion from MCI to AD. To identify biomarkers for MCI and AD, various machine learning methods have been applied, which have improved the prediction and performance and more importantly, the discrimination of patients with MCIs from those MCI patients who will progress to develop AD (MCIc) [12]. Various biomarkers have been identified for the diagnosis of MCI and AD, including functional and structural neuroimaging measures, as well as cognitive score, *APOE ε*4 allele status, and cerebrospinal fluid (CSF) markers. The most recent criteria for AD diagnosis [13] suggest that neuroimaging and biological measures may play a vital role in the early detection of AD and the monitoring of the prodromal stage.

Imaging biomarkers are considered important indicators in the diagnosis of AD and MCI. With the development of neuroimaging technology, structural magnetic resonance imaging (sMRI) techniques have become widely popular and can be used to locate more subtle morphological changes in brain disorders [14, 15]. For dementia patients, MRI is used in the standard clinical assessment. There have been a large number of studies aiming to identify imaging biomarkers for the diagnosis of AD and the prediction of MCI progression. A majority of the well-established structural MRI biomarkers are mainly based on cerebral atrophy measurements or ventricular expansion. Imaging biomarkers, such as cortical thickness [16–19], voxel-wise tissue probability [20–22], and volume [23–25], can show AD-associated atrophy patterns and serve as effective biomarkers to classify AD and MCI. So far to our knowledge, gradual cerebral atrophy is one of the obvious and major changes in AD, and the pattern of atrophy can be analyzed via high-resolution MRI technology. Morphology-related cortical volume, cortical thickness, and cortical area measurements have been utilized to better understand the fundamental pathophysiology of AD diagnosis. However, the majority of these studies [26–28] are mainly focused on differences in cortical and gray matter volumes. Reference to surface area and other biomarkers are still lacking in this regard. The combination of different cortical metrics (i.e., volume, area, and thickness) across multiple brain regions may better distinguish between AD patients and HC. Therefore, advanced machine learning with multivariate approaches, which can establish the subtle relationship between multiple regions and metrics [29, 30], is potentially useful in assisting with prediction and diagnosis of AD. Besides structural changes identified by MRI, other interesting biomarkers for AD detection include CSF components, cognitive score, and the presence of the *APOE ε*4 allele. Numerous CSF, cognitive, and *APOE ε*4 allele studies [12, 31, 32] have been carried out for the classification of AD and MCI. CSF biomarkers that have been utilized in several studies include hyperphosphorylated tau (P-tau), total tau (T-tau), and the A*β*_42_ amino acid. These three CSF components provide valuable information for the identification of AD, as patients have abnormally low levels of A*β*_42_ and high levels of P-tau and T-tau [33]. It has been shown that a combination of T-tau and CSF component measures provides outstanding classification accuracy for separating HC from AD patients, with high sensitivity and specificity [34]. Furthermore, genetic risk factors also impact the imaging and biological markers of AD classification. Several previous studies [35] have shown that the presence of a specific variant of the apolipoprotein E gene (*APOE*) is a crucial risk factor associated with late-onset AD. *APOE* has three majors' alleles: *ε*2, *ε*3, and *ε*4. In comparison with noncarriers, AD patient carriers of the *ε*4 allele typically have low CSF A*β*_42_ and elevated CSF levels of P-tau and T-tau, along with accelerated atrophy patterns on MRI. Various aspects of pathological patterns associated with AD can be revealed by diverse biomarkers; thus, complementary biomarkers might assist diagnosis. It has been shown that a combination of different modalities of biomarkers can enhance diagnostic performance [25, 36–40]. Some recent papers of note [41–43] have demonstrated the feasibility of machine learning approaches. One of the frequently used methods for solving the classification problem is the support vector machine (SVM). A number of studies have applied the SVM for AD prediction and classification [24, 39, 44, 45]. In the field of machine learning, deep learning has gained popularity and become a promising technology. Deep learning relates to multilevel representation learning and abstraction and has resulted in a significant improvement in performance in the field of data analysis and image classification. In recent years, use of deep learning techniques for multimodal data analysis and classification has greatly increased. For example, seamless information is obtained using stacked autoencoders from various types of media [46]. To obtain joint representation of text and images, a multimodal deep belief network was developed [47]. Another study [48] proposed a multisource deep learning method to analyze human pose estimation. A further study [49] developed a modal of combining MRI, positron emission tomography (PET), and CSF modalities using stacked autoencoders to obtain automatic classification of AD. Backpropagation algorithms are used to learn by most deep learning architectures, which iteratively adjust the parameters. For this reason, to reach good generalization performance, conventional neural networks use many iterations [50]. To overcome this situation, Huang et al. [51] proposed an extreme learning machine (ELM), in contrast to traditional methods, with good computational efficiency by randomly assigning weight in the input layer and analytically calculating hidden layer weights. In another study [52], the authors used the dual-tree complex wavelet transform (DTCWT), combined with ELM classifiers, and achieved good accuracy in AD classification. Similarly, in a further study [53], the authors used ELM classifiers with multivariate pattern analysis to classify AD using functional MRI (fMRI) data and achieved outstanding performance. The majority of the current literature regards ELM to be a good machine learning tool [54, 55]. ELM's major strength is that the hidden layer's learning parameters, including the input weights and biases, do not have to be iteratively tuned as in single hidden layer feedforward neural (SLFN) networks. Because of this, ELM costs less and is capable of achieving faster speeds [54]. Furthermore, it is the most favored of in machine learning methods compared to its predecessors. Some of the other commendable attributes of ELM include good generalization accuracy and performance, a simple learning algorithm, improved efficiency, nonlinear transformation during the training phase, possession of a unified solution to different practical applications, lack of local minimal and overfitting, and the need for fewer optimizations, as compared to SVM [55]. Therefore, in this study, we were motivated to use an extreme learning machine to achieve optimum classification accuracy in the identification of AD.

**Figure 1:**
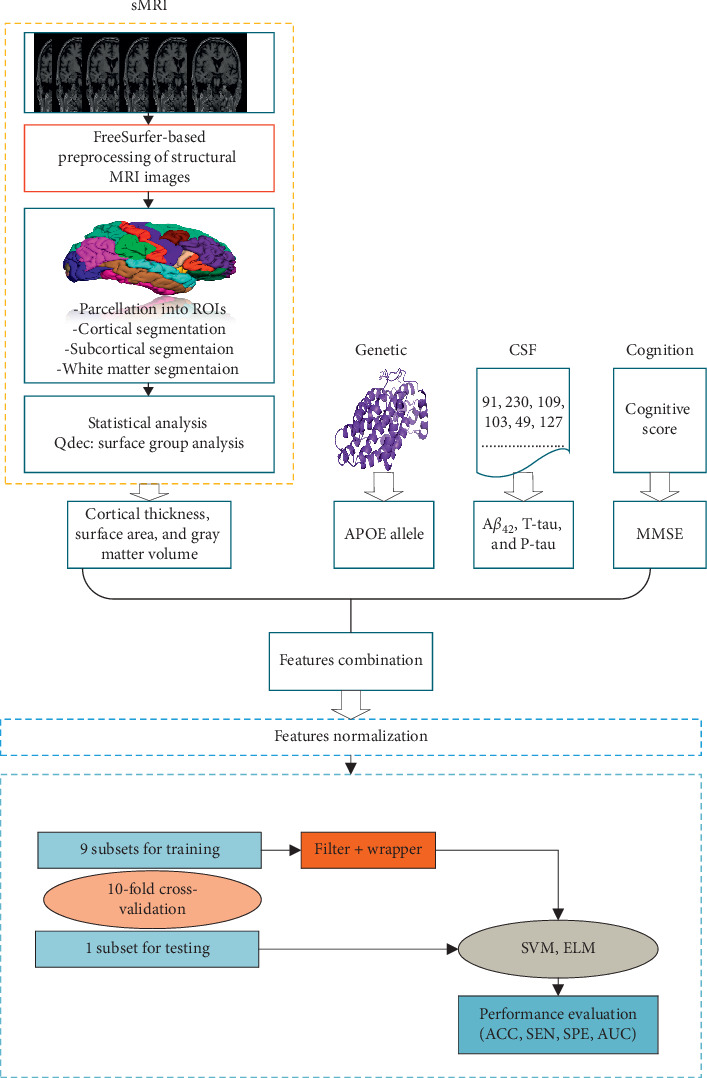
Block diagram of the proposed framework.

Our results, using the Alzheimer's Disease Neuroimaging Initiative (ADNI) dataset (including both MCI patients who did not convert to AD and MCI patients who converted to AD within 36 months), demonstrate the utility of the suggested method. Structural MRI data were firstly preprocessed by FreeSurfer (version 6.0.0) to obtain three types of measures and statistical analysis was performed using query design estimate contrast (QDEC). As well as cortical thickness, and gray matter volume and surface area, we also utilized CSF markers, *APOE ε*4 allele status, and cognitive score. To validate the effectiveness of our method, we compared the classification performance with linear-SVM and RBF-SVM. The general block diagram in Figure 1 shows the workflow of the proposed method.

## 2. Material and Methods

### 2.1. Data

All data used in this analysis were obtained from the ADNI database. The ADNI was initiated in 2003 as public-private partnership, under the Principal Investigator Michael W. Weiner, MD. The primary objective of ADNI is to investigate whether imaging modalities, such as MRI, PET, other neuropsychological assessments, and clinical and biological markers, can be combined to for the early detection of AD and progression of the prodromal state (i.e., MCI). Demographic information, raw neuroimaging data, CSF components, *APOE* genotype, diagnostic information, and neuropsychological test scores are publically available at the ADNI data repository (http://adni.loni.usc.edu). Informed consent was obtained from all participants and the study was approved by the Institutional Review Board of each data site (for more information, see http://adni.loni.usc.edu/wp-content/themes/freshnews-dev-v2/documents/policy/ADNI_Acknowledgement_List%205-29-18.pdf).

For this study, we utilized MRI, CSF, and *APOE* genotype data. The resulting study cohort included patients affected by AD, patients with MCI, and healthy controls. Sociodemographical and clinical information of the participants is shown in Table 1.

### 2.2. Data Acquisition

Structural MRI data were acquired using either Siemens, GE, or Philips scanners at ADNI participating sites. Since the image acquisition protocols varied for each scanner, the image normalization steps were provided by ADNI. Corrections included calibration, geometry distortion, and intensity nonuniformity reduction. Detailed information is available at the ADNI website (http://adni.loni.usc.edu/). These corrections were applied on each MPRAGE image following the image preprocessing steps. In this study, we utilized T1-weighted images, which were collected and reviewed for quality and correction, in terms of data format and alignment. Finally, images with 256 × 256 × 176 resolution and 1 × 1 × 1 mm voxel size were collected.

CSF data were collected in the morning after overnight fasting with the use of a 20- or 24-G spinal needle. Within 1 hour of acquisition, CSF was frozen and transported to the ADNI core laboratory at the Medical Center of Pennsylvania University.

The ADNI biomarker core laboratory also provided genotype and gene expression data for each participant in this study, which were obtained from peripheral blood samples. The genetic feature was a single categorical variable for each participant, taking one of five possible values: (*ε*2, *ε*3), (*ε*2, *ε*4), (*ε*3, *ε*3), (*ε*3, *ε*4), or (*ε*4, *ε*4). In this study, we specifically analyzed *APOE ε*4 allele status (carrier vs. noncarrier).

Cognitive score, obtained from the Mini-Mental State Examination (MMSE) at baseline, was used as the measure of the patient's cognitive performance.

### 2.3. FreeSurfer Analysis of MRI

We applied the recon-all FreeSurfer pipeline (version 6.0.0), which is freely accessible at http://surfer.nmr.mgh.harvard.edu, to sMRI images, for cortical reconstruction and volumetric segmentation [56]. This pipeline automatically generated reliable volume and thickness segmentation of white matter, gray matter, and subcortical volume. Cortical reconstruction and subcortical volumetric segmentation include removal of nonbrain tissues, Talairach transformations, segmentation of subcortical gray and white matter regions, intensity standardization, and Atlas registration. After these steps, a cortical surface mesh model was generated, and finally, the 34 cortical regions were obtained from cortical surface parcellation, based on sulcal and gyral landmarks for both hemispheres corresponding to Desikan et al. [57]. For statistical analysis purposes, smoothing was carried out using recon-all with the qcache option in FreeSurfer. The QDEC tool within FreeSurfer was utilized to analyze differences in cortical thickness, surface area, and gray matter volume between HC, MCI, and AD individuals. Statistical significance levels were corrected for both hemispheres, using the false discovery rate (FDR) *p* < 0.05 to control for multiple comparisons [58].

### 2.4. Machine Learning-Based Prediction and Analysis

An overview of the prediction framework developed for this study is shown in Figure 1. The framework consists of four major steps: feature extraction, feature combination, normalization, and feature selection and classification. We used two machine learning classification algorithms, SVM and ELM.

### 2.5. Feature Selection

The feature selection algorithm is an essential part of a machine learning approach, facilitating data understanding, reducing storage requirements and training-testing times, and improving the accuracy of classification. Importantly, feature selection was performed using only the training dataset and then applied on the test set. Before feature selection, we performed feature normalization, and all feature sets were normalized to unit variance and zero mean to reduce redundancy and improve data integrity between the feature sets. For a given data matrix *X*, columns represent features and rows represent the participants' normalized matrix *X*_norm_ with an element (*i*, *j*), which was calculated as shown in equation (1). After feature normalization, we used a combination of filter and wrapper algorithms for feature selection. We used a filter method and sorted features based on their minimum redundancy maximum relevance (MRMR) scores. MRMR has been previously described [59]. The MRMR score for a feature set *S* is defined in equation (2):

(1) $$\begin{array}{c} X_{\text{norm}}=\frac{x_{\left(i,j\right)}-\text{mean}\left(X_{j}\right)}{\text{std}\left(X_{j}\right)}, \end{array}$$

(2) $$\begin{array}{c} \text{MRMR}={\text{MAX}}_{s}\left\{\frac{1}{\left|s\right|}\sum_{f_{i}\in S}I\left(f_{i};c\right)-\frac{1}{{\left|S\right|}^{2}}\sum I\left(f_{i};f_{j}\right)\right\}, \end{array}$$

where the relevance of a feature set *S* for *k* classes *C*={*c*_1_, *c*_2_,…*c*_*k*_} is defined by the average value of mutual information between the individual feature *f*_*i*_ and *C*, and the redundancy of all features in the feature set *S* is the average value of mutual information between features *f*_*i*_ and *f*_*j*_. The top 60 features identified by the MRMR algorithm were used in a wrapper algorithm to find an optimal subset of features. We developed and validated a sequential feature selection (SFS) algorithm as a wrapper feature selection method for this study. The SFS algorithm has been previously described in detail [60]. Briefly, different subsets of features were selected from the top 60 features identified by the MRMR algorithm, and then the accuracy of the ELM classifiers based on these subsets of features was calculated.

### 2.6. SVM Classifier

Generally, the SVM [61] is a binary classifier, which is applicable to both separable and nonseparable datasets. It has been successfully utilized in the neuroimaging field and has become the most popular machine learning algorithm in the field of neuroscience during the past decade. The SVM is a supervised classifier that uses a training dataset to find an optimal separating hyperplane in an *n*-dimensional space. The optimal hyperplane is one that best separates the two target participant groups. In our study, we utilized both linear SVM and nonlinear SVM based on radial basis function (RBF) kernels. An RBF kernel performs better than a linear kernel for a small number of feature sets. A regularization constant *C* and a set of kernel hyperparameters *γ* (gamma) need to be tuned in SVMs. These parameters were optimized using a cross-validation (CV) method. This procedure was repeated 1000 times, each time randomly selecting a new set of 10 held-out participants to obtain optimum hyperparameters optimization. In this method, the search scales for regularization constant and gamma values were set to *C* = (0.001, 0.01, 0.1, 1, 10, 100, 1000) and *γ* = (0.001, 0.01, 0.1, 1, 10, 100, 1000), respectively. The maximum validation accuracy was obtained at *C* = 1 and *γ* = 0.1. The tuned parameters were used to predict the accuracy value on the test dataset.

### 2.7. ELM Classifier

The extreme learning machine is composed of a hidden layer in between the input and output layers [51]. Whereas weights and biases are required to for adjustment by gradient-based learning algorithms on traditional feedforward neural networks for all layers, in the ELM hidden layer biases and input weights are arbitrarily assigned without iterative processes, and output weights are computed by solving a single hidden layer system [50]. Thus, compared to traditional neural networks, the ELM learns much faster and it is widely used in various regression and classification tasks, being an efficient and reliable learning algorithm [62–65]. Particularly, for *N* training samples {(*X*^(*j*)^, *I*^(*j*)^)|*X*^(*j*)^ ∈ ℛ^*p*^ and *I*^(*j*)^ ∈ ℛ^*q*^, and *j*=1,  2,…, *N*}, the output in ELM, *o*_*j*_ with *n*_*h*_ hidden neurons can be represented as shown in the following equationfd3:

(3) $$\begin{array}{c} o_{j}=\sum_{i=1}^{n_{h}}\beta_{i}^{T}a\left(w_{i}^{T}X^{\left(j\right)}+b_{i}\right)=\sum_{i=1}^{n_{h}}\beta_{i}^{T}h_{i}\left(X^{\left(j\right)}\right)=h{\left(X^{\left(j\right)}\right)}^{T}\beta , \end{array}$$

where *X*^(*j*)^ and *I*^(*j*)^ are the *j*^th^ input and target vectors, respectively. The parameters *p* and *q* are the input and target vector dimensions, respectively. Additionally, *o*_*j*_ ∈ ℛ^*q*^ signifies the output of the ELM for the *j*^th^ training sample, *w*_*i*_ ∈ ℛ^*p*^ indicates the input weight that links the input nodes to the *i*^th^ hidden node, *b*_*i*_ represents the bias of the *i*^th^ hidden node, and *a*(·) signifies the activation function for the given hidden layer. *β*=[*β*_1_,…, *β*_*n*_*h*__]^*T*^ are the values of the output weights between the output neurons, and the hidden layer *h*(*X*^(*j*)^)=[*h*_1_(*X*^(*j*)^,…, *h*_*n*_*h*__(*X*^(*j*)^))]^*T*^ is the output vector of the hidden layer with respect to the *j*^th^ training sample *X*^(*j*)^ · *h*_*i*_(*X*^(*j*)^) is the output of the *i*^th^ hidden layer for the *j*^th^ training sample. To obtain the optimal hidden layer weights, $\hat{\beta }$ with respect to *N* training samples can be considered to solve the following optimization problem:

(4) $$\begin{array}{c} \;\underset{\beta }{\min}\;\lambda \;{\left\|H\beta -L\right\|}^{2}+{\left\|\beta \right\|}^{2}, \end{array}$$

where *H*=[*h*(*X*^(1)^,…,*h*(*X*^(*N*)^))]^*T*^ and *L*=[*I*^(1)^,…,*I*^(*N*)^]^*T*^.

Equation (4) represents the optimization problem, and its optimal solution $\hat{\beta }$ can be analytically obtained as follows:

(5) $$\begin{array}{c} \hat{\beta }=H^{T}{\left(\frac{1}{\lambda }I+HH^{T}\right)}^{-1}L, \end{array}$$

where *λ* is a regularization parameter and *I* represents the identity matrix. After finding the optimal solution $\hat{\beta }$, the output of the ELM on test data *X*_test_ is determined as follows:

(6) $$\begin{array}{c} o_{\text{test}}=h{\left(X_{\text{test}}\right)}^{T}H^{T}{\left(\frac{1}{\lambda }I+HH^{T}\right)}^{-1}L. \end{array}$$

In this proposed method, the hidden node number was set between 1 and 500, and we selected a sigmoid as an activation function. Further, we used a grid search method to tune the ELM parameter on the training dataset in order to achieve optimum cross-validated validation accuracy. Similarly, to minimize the random effects during the weight initializations, each parameter of the number of hidden nodes was used 100 times and the average performance was calculated.

### 2.8. Cross-Validation and Performance Evaluation

We used the *k*-fold cross-validation (KCV; *k* = 10) method for cross-validation. All participants were randomly divided into 10 equally sized subsets using the KCV (*k* = 10) cross-validation approach. In each fold of the KCV, 90% of the data were used to train the model based on a subset of features, and then 10% of the data (cross-validation set) were used to calculate the accuracy of the ELM classifier. Accuracies of the ELM classifiers corresponding to all subsets of features were calculated and classified with maximum accuracy, and the corresponding optimal subset of features was identified. Similarly, classification performance was evaluated on accuracy (ACC), specificity (SPE), and sensitivity (SEN). TP, FP, FN, and TN represent the number of true positives, false positives, false negatives, and true negatives, respectively. In terms of numerical values, ACC, SPE, and SEN can be calculated as follows:

(7) $$\begin{array}{c} \text{accuracy}\left(\text{ACC}\right)=\frac{\left(\text{TN}+\text{TN}\right)}{\left(\text{TP}+\text{TN}+\text{FP}+\text{FN}\right)}, \end{array}$$

(8) $$\begin{array}{c} \text{sensitivity}\left(\text{SEN}\right)=\frac{\left(\text{TP}\right)}{\left(\text{TP}+\text{FN}\right)}, \end{array}$$

(9) $$\begin{array}{c} \text{specificity}\left(\text{SPE}\right)=\frac{\left(\text{TN}\right)}{\left(\text{TN}+\text{FP}\right)}. \end{array}$$

The other effective way to evaluate results for a classifier is the receiver operating characteristic (ROC) curve. The ROC curve is the plot of true-positive rate against false-positive rate by changing the discrimination threshold and therefore summarizing the classifier's performance. The ROC curve is usually represented by the area under the curve (AUC), which is denoted by a number between 0 and 100.

## 3. Results and Analysis

### 3.1. Statistical Analysis

**Figure 2:**
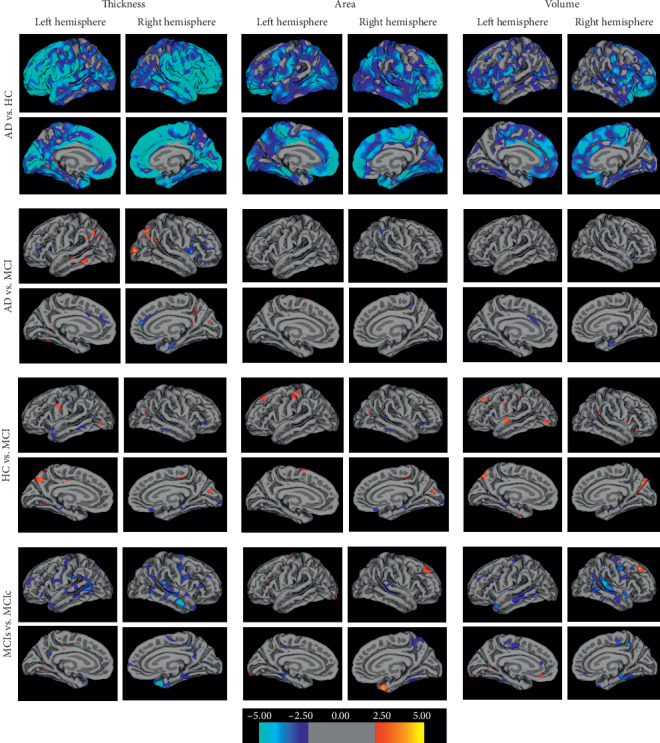
Differences in cortical thickness, area, and volume in patients at different stages of Alzheimer's disease. The colored bar represents the significance level of clusters. The significance threshold was set at *p* < 0.05.

Cortical thickness, gray matter (GM) volume, and surface area were analyzed using a surface-based group analysis in FreeSurfer's QDEC (version 1.5). First, the spatial cortical thickness, GM volume, and surface area of both hemispheres were smoothed with a circularly symmetric Gaussian kernel of 10 mm full-width half-maximum, to normally distribute the results. Then we employed a general linear model (GLM) analysis with age, sex, and education as the nuisance factors in the design matrix to directly compare the three parameters in both hemispheres of the AD vs. HC, HC vs. MCI, AD vs. MCI, and MCIs vs. MCIc groups. Statistical analysis results regarding cortical thickness, surface area, and gray matter volume are shown in Figure 2. The Desikan–Killiany Atlas divides the human cerebral cortex into 34 cortical regions in each hemisphere. As there were a high number of atrophic regions, we present only the top-ranked regions with significant differences. The atrophic regions for three parameters are listed below.

Table 2 presents the atrophy position and range of clusters for the differences in gray matter volume, cortical thickness, and surface area at each vertex between HC, MCI, and AD groups by QDEC analysis. In this table, only the top features which have significant cluster differences for each kind of parameter are provided. From the statistically significant brain regions shown in Figure 2 and Table 2, we observe the following:

1. The cortical thicknesses of the left insula, left cuneus, paracentral, right rostral middle frontal, and right pars opercularis areas were thinner in the AD group compared with the HC group. For HC vs. MCI, the cortical thicknesses of the left precuneus, left lingual, left and right insula, right pars triangularis, and right inferior parietal areas were thinner. Similarly, for AD vs. MCI, the cortical thicknesses of the left inferior parietal, right lateral occipital, right inferior parietal, and left and right superior temporal areas showed the most atrophy. For MCIs vs. MCIc, the cortical thicknesses of the left inferior parietal, left parahippocampal, right temporal pole, and right superior temporal areas showed the greatest differences.
2. Regarding surface area, the AD group had smaller values than the HC group in the left and right paracentral, left lateral orbitofrontal, right inferior parietal, right posterior cingulate, and right inferior parietal areas. For HC vs. MCI, the left superior frontal, left postcentral, left superior parietal, right supramarginal, right fusiform, right precuneus, and right precentral areas showed the lowest values. Similarly, for AD vs. MCI, the left superior parietal, left and right precentral, left paracentral, right inferior parietal, and right superior frontal areas showed the largest decreases in surface area. For MCIs vs. MCIc, the left fusiform, left lateral occipital, left parahippocampal, right superior parietal, and right postcentral areas showed the most differences.
3. In comparison with the HC group, the volume of gray matter of the left caudal anterior cingulate, left orbitalis, left lateral orbitofrontal, right lateral orbitofrontal, right rostral middle frontal, and right pars opercularis areas was lower in the AD group. For AD vs. MCI, the left superior parietal, left superior frontal, left caudal anterior cingulate, right entorhinal, and right superior parietal areas showed the largest decreases in volume. For HC vs. MCI, the left supramarginal, left cuneus, left precentral, right parahippocampal, and right superior parietal areas showed the most volume atrophy. Similarly, for MCIs vs. MCIc, the left bankssts, left precentral, left rostral middle frontal, right precentral, and right fusiform areas had the largest decreases in volume.

Moreover, from this analysis, we observe that area thickness and volume in the AD group were significantly decreased in comparison with the HC group. Similarly, there was significant atrophy in the MCIc group in comparison with the MCIs group, and there was little difference in the atrophy pattern among AD and MCI patients.

### 3.2. Feature Selection and Classification

**Figure 3:**
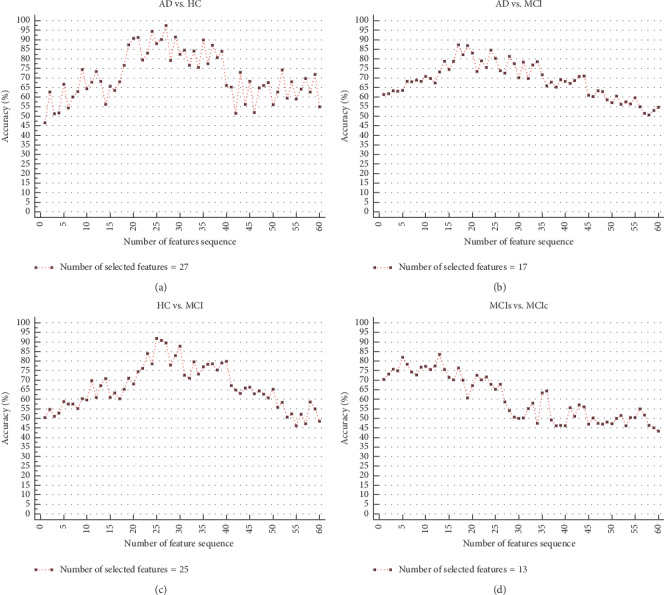
Number of feature sets selected from the sequential feature selection algorithm on the top 60 ranked features obtained using the MRMR algorithm. Only the number of features versus accuracy for the combination of all feature sets using ELM classifiers is shown. (a) AD vs. HC feature subset. (b) AD vs. MCI feature subset. (c) HC vs. MCI feature subset. (d) MCIs vs. MCIc feature subset.

The individual feature set was selected using an MRMR (filter) and a sequential feature selection (wrapper) method to identify the optimal feature set for different groups and improve the classifier accuracy. Similarly, we combined all the feature sets from different measures and applied the MRMR and SFS method to select the optimal features. Multiple measures feature sets were created by combining cortical thickness, area, and volume from sMRI, three component measurements from CSF, *APOE ε*4 status, and MMSE score. First, we selected the top 60 features from the MRMR algorithm based on the features score, and then we applied a sequential feature selection algorithm on these top 60 ranked features, which gave the optimal feature set to achieve the maximum classification accuracy on ELM classifiers.  Figure 3 shows the number of optimal features sequence after sequential feature selection on the top 60 ranked features obtained from the MRMR algorithm using ELM classifier.  Figure 3 shows only the selected features for the combined feature set. From this, we can assume that the proposed feature selection with cross-validation provides the optimal feature vector for input to the classifiers. In this proposed method, classification performance was quantified by the number of features selected versus accuracy and area under the ROC curve.

**Figure 4:**
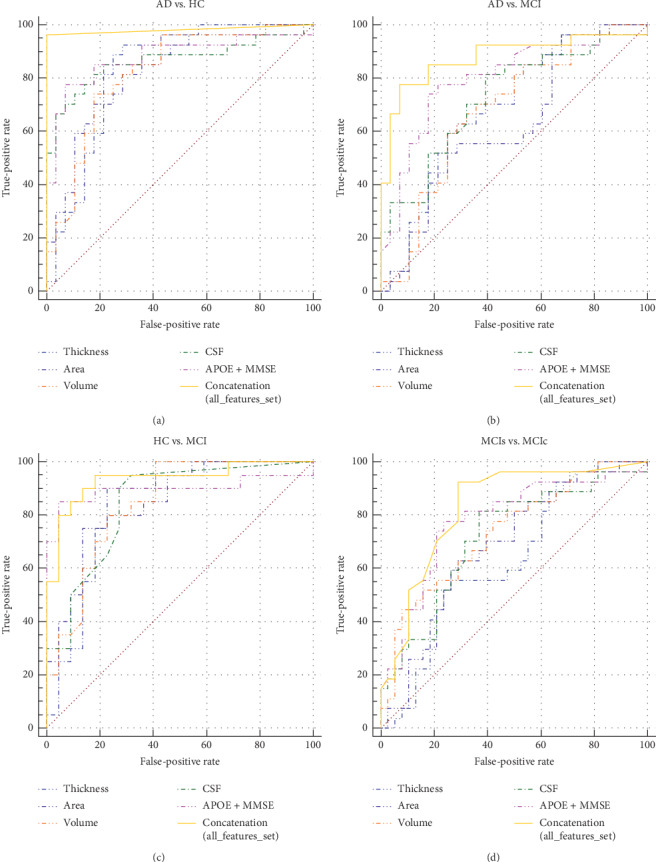
ROC curves for discriminating AD, MCIs, MCIc, and HC (healthy controls). ROC curves are plotted for the five biomarkers separately and for the concatenation of all features (i.e., all five biomarkers measures). (a) ROC curves for AD vs. HC classification, (b) for AD vs. MCI classification, (c) for HC vs. MCI classification, and (d) for MCIs vs. MCIc classification, using two-stage feature selection with ELM classifiers.

To further analyze the effectiveness of the purpose classification method combining different measures, we calculated the AUCs for the concatenation of all features.  Figure 4shows the receiver operating curves for individual features and all feature (imaging and nonimaging biomarkers, i.e., multiple features) combinations for each classification group, using the ELM classifier.

## 4. Discussion

### 4.1. Performance Analysis

In this study, we first performed statistical analysis and pattern classification to differentiate and identify atrophy patterns for the four groups (AD, HC, MCIs, and MCIc). Individual sMRI was preprocessed using the FreeSurfer tool. After preprocessing the statistical analysis results of sMRI, QDEC was applied and finally we performed the classification task by the proposed feature selection and classification method, respectively. For brain atrophy analysis, we used three types of sMRI cortical metrics (cortical thickness, gray matter volume, and surface area). In comparing the AD group with the HC group, the insula, pars opercularis, parahippocampal, and superior temporal areas were severely affected in terms of cortical thickness, gray matter volume, and surface area. The cortical thickness of the left hemisphere was thinner than that of the left hemisphere. Similarly, for AD vs. MCI, the most atrophy was seen in the left inferior parietal and right lateral occipital areas. For HC vs. MCI, the supramarginal and cuneus areas showed the most atrophy. For MCIs vs. MCIc, the superior temporal, and precentral areas showed the largest decreases in thickness, volume, and area. Based on these differences, the majority of the decrease in cortical thickness, gray matter volume, and surface area appears mostly in the frontal lobe, temporal lobe, occipital lobe, cingulate gyrus, and parietal lobe. This phenomenon strongly agrees with findings related to atrophy patterns seen in previous studies [14, 66]. These regions are mainly involved in the expression of personality, motor execution, complex cognitive behavior, and decision making [50]. In addition, we present the less common analysis of MCIs vs. MCIc. For MCIc, the most atrophy was seen in the superior temporal, bankssts, precentral, inferior parietal, and insula areas, which show potential for the early recognition of progression to Alzheimer's disease.

**Figure 5:**
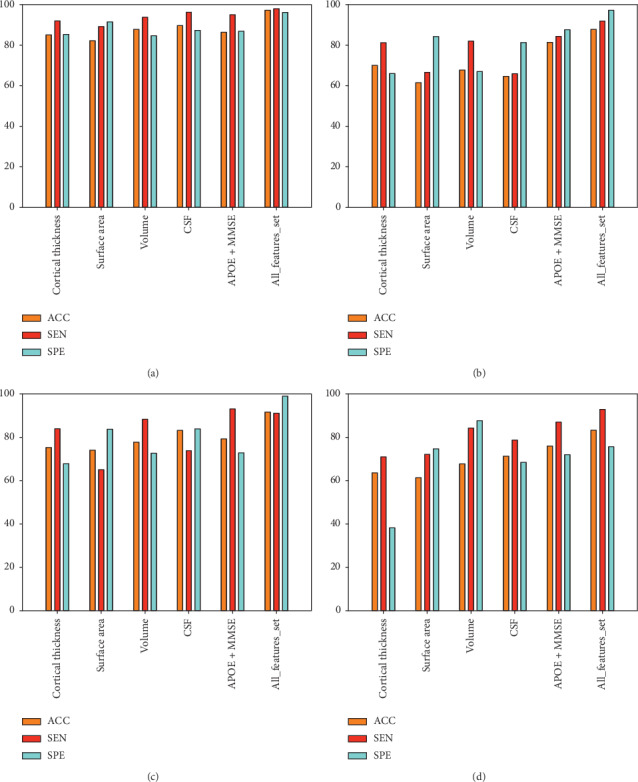
Classification results for different Alzheimer's groups using ELM classifiers. (a) Classification results for AD vs. HC groups, (b) for AD vs. MCI groups, (c) for HC vs. MCI groups, and (d) for MCIs vs. MCIc groups.

**Figure 6:**
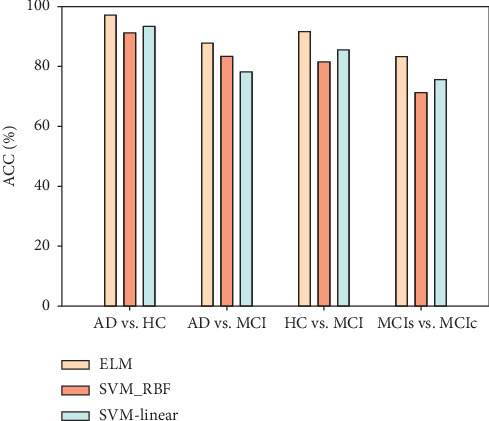
Performance comparison of different classifiers.

To test the effectiveness of our purposed features combination method (i.e., imaging and nonimaging features), we adapted the individual feature from sMRI and CSF separately to conduct the study, although, regarding genetic and cognitive features, we combined them to test the performance and then compared the accuracy with the accuracy of the combination of all features. For the proposed feature selection, we used a combination of filter and wrapper algorithms and compared the performance of the classifiers on the selected feature set. In this text, SVM-linear, SVM-RBF, and ELM were used for classification. The classification results for the compared methods are presented in Tables 3–6 and also in graphical form in Figure 5. As shown in the tables, the performance accuracy of feature fusion is noticeably improved when compared with that of the individual feature set, and there are distinct degrees of elevation in other indexes; the specificity index is particularly more noticeable. In contrast, performance accuracy based on the nonimaging features is almost the same as the imaging features for AD diagnosis, but far superior for MCI diagnosis. For AD vs. HC, the accuracy obtained by CSF measures, genetics, and cognitive score showed a lower increase, although for MCI vs. HC, AD vs. MCI, and MCIs vs. MCIc, the accuracy was higher. The result showed that the ELM classifier achieves better classification scores compared to the SVM classifier (linear and RBF-SVM), for both single modality feature sets, as well as all features combined (shown in Figure 6). The ELM is good machine learning tool, and the major strength is that the hidden layer's learning parameters, including input weight and biases, do not tune iteratively, as in SLFN. The ELM offers many advantages over other learning algorithms [54]. From the results shown in Tables 3–6, for AD vs. HC, the classification result increased by 5–7% as compared to SVM classifiers, with 98.04% sensitivity and 96.28% specificity. For AD vs. MCI, classification accuracy increased by 5–9% with 92% sensitivity and 97.33% accuracy. For HC vs. MCI, classification accuracy increased by 6–10% with 91.23% sensitivity and 99.13% specificity. Similarly for MCIs vs. MCIc, classification accuracy increased by 7–12% with 93.01% sensitivity and 75.77% specificity, but 84.67% and 76.67% specificity was obtained for linear and RBF-SVM classifiers, respectively, which is more than obtained by the ELM classifier. From our analyses, we believe that the ELM gives better performance as compared to SVM from a learning efficiency standpoint because the ELM's original design has high learning accuracy, fast learning speed, scalability, and the least human intervention [54, 55]. From the results shown in Table 7, we can see that our suggested method achieved better accuracy than other existing methods. For classifying AD and HC, our method achieved a classification accuracy of 97.31%, with a sensitivity of 98.04%, a specificity of 96.28%, and an AUC value of 0.97%. For classifying MCI and HC, our method achieves a classification accuracy of 91.72%, with a sensitivity of 91.23%, a specificity of 99.13%, and an AUC value of 0.93%. For distinguishing AD from MCI, our proposed method obtained a classification accuracy of 87.91%, with a sensitivity of 92.00%, a specificity of 97.33%, and an AUC value of 0.89%. Similarly for MCIs vs. MCIc, we achieved outstanding performance as compared to previous methods by combining multiple features, with an accuracy of 83.38%, a sensitivity of 93.01%, a specificity of 75.77%, and an AUC value of 0.85%.

### 4.2. Comparison with Other Methods

In the previous section, we discussed in detail the findings of the proposed feature selection and classification method. In this section, we compare and discuss the findings of our method in comparison with existing state-of-the-art methods. Tables 7 and 8 show a comparison of the classification performance of the proposed method with recently published studies which used multimodality data to distinguish individuals with AD and MCI from HC. Westman et al. [67] used MRI and CSF biomarkers and obtained 91.8% accuracy for AD vs. HC, 77.6% for HC vs. MCI, and 68.5% for pMCI vs. MCIs classification using the orthogonal partial least squares to latent structures (OPLS) method. Zhang and Shen [39] used a multimodal (MRI, PET, and CSF) classification method with multitask feature selection and an SVM classifier for AD and MCI classification. By combining MRI, PET, and CSF data, they achieved a higher accuracy of 93.3% for AD vs. HC and 83.2% accuracy for HC vs. MCI classification. Similarly, the authors achieved an accuracy of 73.9% on pMCI vs. MCIs samples. Hinrichs et al. [69] obtained an accuracy of 92.4% for AD vs. HC classification using MRI, PET, CSF, *APOE*, and cognitive score in multiple kernel learning. Similarly, Johnson et al. [68] proposed a sparse representation learning feature selection and stacked autoencoder for classifying AD using MRI, PET, CSF, and cognitive scores as features and obtained 95.9% accuracy for AD vs. HC classification and 85% accuracy for HC vs. MCI classification. Maqsood et al. [72] proposed the transfer learning classification model of AD for both binary and multiclass problems. The algorithm utilizes a pretrained AlexNet network and fine-tuned the convolutional neural network (CNN) for classification. This model was fine-tuned over both segmented and unsegmented 3D views of the human brain. The MRI scans were segmented into the characteristic components of GM, white matter, and CSF. The retrained CNN was then validated using the test data to obtain accuracies of 89.6% and 92.8% for binary and multiclass problems, respectively, using the OASIS dataset. Beheshti et al. [70] developed a computer-aided diagnosis (CAD) system composed of four systematic stages for analyzing global and local differences in the GM of AD patients, compared with HC using the voxel-based morphometry (VBM) method. They used feature ranking based on the *t*-test and a genetic algorithm, with Fisher's criteria as part of the objective function in the genetic algorithm. The authors utilized the SVM classifier with 10-fold cross-validation to obtain accuracies of 93.01% and 75% for AD vs. HC and MCIs vs. pMCI classification, respectively. Spasov et al. [71] developed a deep learning architecture, based on dual learning and an ad hoc layer for 3D separable convolution to identify MCI patients. Their deep learning procedure combined MRI, neuropsychological, demographic, and *APOE ε*4 data to achieve an accuracy of 86%. They achieved an accuracy of 99.5% for AD vs. HC classification. In another study, Moradi et al. used VBM analysis of GM as a feature combined with age and cognitive measures. They achieved an accuracy of 82% for pMCI vs. MCIs classification. From this comparison, we can infer that the proposed feature combination (MRI, CSF, *APOE,* and MMSE data) is robust or comparable to the other multimodal biomarker methods reported in the literature, for both AD vs. HC and MCIs vs. MCIc classification.

## 5. Conclusion

In conclusion, we demonstrated that a combination of three sMRI measures, cortical thickness, cortical area, cortical volume, and three nonimaging measures, CSF components, *APOE ε*4 status, and MMSE score, improves AD diagnosis. Furthermore, this combination shows great potential for the early identification of mild cognitive impairment (the prodromal stage of Alzheimer's disease). In this method, we proposed filter and wrapper feature selection with an ELM classifier for multiple biomarker-based AD diagnosis, which significantly improved the classifier's performance. Moreover, the results were better than, or comparable with, those previously reported, particularly for the most challenging classification, such as HC vs. MCI and MCIs vs. MCIc. The added value of combining different anatomical MRI measures should be considered in AD scanning protocols. Only using specific aspects, or a single measure of whole brain atrophy, for AD diagnosis is still common practice. Our results show that clinical AD diagnosis could benefit from the combination of multiple measures from an anatomical MRI scan, and other nonimaging biomarkers, incorporated into an automated machine learning system. Our suggested method effectively enhances the diagnostic accuracy of AD and MCI, but still has some drawbacks. Future work will include various improvements. First, we will optimize the parameter obtaining process. Second, in order to enhance the effectiveness of the suggested method, the dataset will be increased in terms of the following: extension of the longitudinal dataset for better understanding of the progression from MCI and inclusion of multimodal data, such as PET and fMRI data, which provides different insights into the characteristics of AD.

## Figures and Tables

**Table 1:** Baseline clinical and sociodemographical information of the participants of this study.

Group	AD	MCI	HC	MCIs	MCIc
No. of participants	53	77	57	35	42
Female/male	20/33	34/43	32/25	13/22	21/21
Age	74.4 ± 7.8	74.1 ± 7.2	75.6 ± 5.2	73.9 ± 7.2	74.3 ± 7.2
Education	15.1 ± 3.2	15.9 ± 2.9	15.7 ± 2.8	16.1 ± 2.9	15.8 ± 2.9
MMSE	23.5 ± 1.8	26.9 ± 1.8	29.1 ± 0.9	27.2 ± 1.7	26.6 ± 1.8
CDR	0.7 ± 0.2	0.5	0	0.5	0.5

The entries for age, gender, education, and MMSE denote mean and standard deviation for each group. MMSE, Mini-Mental State Exam; CDR, clinical dementia ratio.

**Table 2:** Cluster differences in cortical thickness, area, and volume in AD, MCI, and MCIc patients.

Feature	Region	Coordinates			Vertex	Value	Size (mm^2^)
		*x*	*y*	*z*			
AD vs. HC							
Thickness	Left insula	−29.5	17.9	11.9	3165	−2.3512	3619.47
	Left parahippocampal	−31.3	−41.7	−8.7	557	−2.2303	10736.27
	Left cuneus	−47.9	−20.9	42.8	1039	−2.3188	2824.28
	Right rostral middle frontal	38.5	43	7	1224	−2.1737	14.8
	Right superior temporal	53.3	−5.2	−5.2	468	−2.8676	256.28
	Right pars opercularis	50.3	10.2	8.8	1242	−3.6071	58155.25
Area	Left paracentral	−15.8	−35.5	49.4	1197	−2.2767	7018.72
	Left lateral orbitofrontal	−53.3	−2	7.5	489	−2.1962	3218.23
	Right paracentral	12.5	−37.1	55.4	1233	−2.2555	42.55
	Right inferior parietal	34	−51.9	37.4	1133	−2.1026	7213.05
	Right posterior cingulate	14	−30.4	36.6	1201	−2.0979	1416.67
	Right inferior parietal	39.5	−44.7	34.6	7	−2.0263	50.76
Volume	Left caudal anterior cingulate	−5	18.4	26.4	2073	−3.2264	834.61
	Left orbitalis	−28.7	−60.2	41.7	1103	2.3437	1202.55
	Left lateral orbitofrontal	−59.4	−6.9	9.4	412	−2.0146	635.67
	Right lateral orbitofrontal	30.4	22.9	−20.3	631	−2.7411	2025.6
	Right rostral middle frontal	34.8	51	4.7	198	−2.717	1098.82
	Right pars opercularis	46.8	15.2	8.7	136	−2.717	8077.1

AD vs. MCI							
Thickness	Left inferior parietal	−43.6	−61.5	33	939	2.7022	439.37
	Left fusiform	−40	−53.7	−20.3	254	2.459	149.84
	Left superior temporal	−47.7	−25	−9.1	210	−2.5577	328.03
	Right lateral occipital	26.1	−93.9	3.7	363	3.4944	363
	Right inferior parietal	37.7	−71.7	42.8	1129	3.3654	646.24
	Right superior temporal	50.7	−14.3	−2.4	722	−2.9715	342.28
Area	Left superior parietal	28.4	−51	42.7	1702	−2.5085	642.76
	Left precentral	32.2	−21.8	60.8	109	1.8969	51.54
	Left paracentral	14.1	−36.7	52.4	238	−1.8959	79.68
	Right inferior parietal	−35.2	−87.2	13.7	50	−1.8099	34.53
	Right precentral	−54.2	−1.1	7.1	51	−1.7244	21.34
	Right superior frontal	−7.4	4	66.5	34	1.6541	17.86
Volume	Left superior parietal	−28.5	−58.8	40	540	3.5868	216.1
	Left superior frontal	−8.5	41.2	30.3	48	−2.6334	33.98
	Left caudal anterior cingulate	−4.9	11.3	32.1	314	−2.549	150.07
	Right entorhinal	21.5	−8.9	−29.5	207	−3.1433	55.69
	Right lateral orbitofrontal	42.8	27.8	−13.7	198	−3.1034	125.01
	Right superior parietal	32.1	−44.2	41.2	115	−2.5309	40.29

HC vs. MCI							
Thickness	Left insula	−36.4	−9.4	−11.7	811	−3.5988	309.94
	Left precuneus	−6	−68.9	41.7	628	2.3682	322.02
	Left lingual	−29.3	−44.5	−6.6	612	−2.7175	279.16
	Right insula	35.8	−10.6	−7.4	1154	−3.1385	436.62
	Right pars triangularis	38.9	31.7	1	372	−2.1264	204.87
	Right inferior parietal	35.1	−72	40.9	285	−2.7121	152.2
Area	Left superior frontal	−17.8	31.5	49.6	237	1.7293	156.88
	Left postcentral	−52.1	−22.7	50.7	264	1.7286	122.75
	Left superior parietal	−32	−49.3	47.3	141	−1.7572	66.04
	Right supramarginal	53.4	−47.4	35	1336	2.7163	676.85
	Right fusiform	34.7	−12	−34.1	551	2.0892	322.84
	Right precuneus	18.2	−77.3	27.7	482	1.7491	313.36
	Right precentral	20.7	−30.6	53.9	328	−1.9165	115.45
Volume	Left supramarginal	52.1	−46.3	22.6	441	2.7521	210.23
	Left cuneus	17.1	−69.5	16.8	628	2.611	481.74
	Left precentral	21.2	−30.7	53.8	376	−2.1546	127.02
	Right parahippocampal	−23.8	−36.1	−15.6	278	−1.8212	127.68
	Right superior parietal	−9.1	−74.3	46.5	598	2.5199	303.67
	Right superior frontal	−18.1	33.3	39.6	210	2.6035	114.72

MCIs vs. MCIc							
Thickness	Left inferior parietal	−46.3	−60	11.1	2770	−3.7613	1427.48
	Left superior temporal	−45.5	−0.4	−20.8	1067	−2.6742	466.24
	Left parahippocampal	−31.7	−40.3	−10.1	550	−2.3925	239.85
	Right temporal pole	29.2	9	−38	1449	−5.5983	822.82
	Right superior temporal	62.3	−34.7	15.2	1292	−3.1646	549.97
	Right inferior temporal	51.6	−56.6	−3.7	882	−3.0694	507.91
	Right precentral	48.8	−6.5	40.5	858	−2.8315	351.93
Area	Left fusiform	−36.4	−29.5	−22	659	−3.0958	312.21
	Left lateral occipital	−19.4	−99	−15.1	326	2.6022	250.61
	Left parahippocampal	−18.8	−33.5	−14	224	−2.0347	97.59
	Right superior temporal	48.1	−32.9	2.2	1515	−2.5815	579.8
	Right superior parietal	10.6	−52.7	65.1	1697	−2.2367	641.52
	Right postcentral	33.8	−29	51.9	799	−2.0859	367.23
Volume	Left bankssts	−57.9	−46.9	−1	2196	−2.6263	1162.58
	Left precentral	−36.1	−22	51.7	2736	−2.5339	1073.1
	Left rostral middle frontal	−22.4	27.9	32.8	582	−2.3081	326.86
	Right superior temporal	62.3	−34.7	15.2	3392	−4.4677	1449.96
	Right precentral	49.5	−6.2	41.1	4103	−3.2472	1813.94
	Right fusiform	33.9	−37.8	−22.8	782	−3.1822	425.05

**Table 3:** 10-fold cross-validated classification performance for AD vs. HC.

Features measure	ELM				SVM-RBF			SVM-linear		
	ACC%	SEN%	SPE%	AUC	ACC%	SEN%	SPE%	ACC%	SEN%	SPE%
Cortical thickness	85.13	92.07	85.44	0.86	80.68	85.68	83.81	77.17	83.17	73.81
Surface area	82.30	89.30	91.54	0.85	76.16	86.16	75.14	78.67	87.67	72.62
Volume	87.90	93.87	84.75	0.88	79.29	74.29	78.33	80.00	77.10	63.24
CSF	89.73	96.33	87.38	0.89	79.05	89.05	74.17	75.33	83.33	68.57
APOE + MMSE	86.45	95.13	87.00	0.93	82.03	87.03	84.67	84.16	83.05	78.79
Concatenation (all_features_set)	97.31	98.04	96.28	0.97	91.33	93.33	87.57	93.50	95.5	90.58

**Table 4:** 10-fold cross-validated classification performance for AD vs. MCI.

Features measure	ELM				SVM-RBF			SVM-linear		
	ACC%	SEN%	SPE%	AUC	ACC%	SEN%	SPE%	ACC%	SEN%	SPE%
Cortical thickness	70.10	81.30	66.10	0.71	64.01	83.83	62.33	65.03	78.80	73.30
Surface area	61.60	66.70	84.30	0.63	63.45	68.10	80.11	60.67	71.23	62.85
Volume	67.80	82.10	67.10	0.69	58.20	77.92	68.56	60.05	73.37	63.73
CSF	64.70	66.00	81.40	0.73	56.07	62.75	75.73	61.23	68.71	79.37
APOE + MMSE	81.45	84.40	87.70	0.82	76.81	67.81	85.48	76.20	66.94	87.48
Concatenation (all_features_set)	87.91	92.00	97.33	0.89	83.50	88.64	76.72	78.31	74.45	82.62

**Table 5:** 10-fold cross-validated classification performance for HC vs. MCI.

Features measure	ELM				SVM-RBF			SVM-linear		
	ACC%	SEN%	SPE%	AUC	ACC%	SEN%	SPE%	ACC%	SEN%	SPE%
Cortical thickness	75.40	84.07	67.90	0.83	74.17	80.81	71.23	67.34	73.17	77.93
Surface area	74.15	65.20	83.85	0.84	63.54	67.67	66.47	68.72	74.43	67.35
Volume	77.84	88.41	72.80	0.84	65.47	74.73	68.75	72.83	78.27	65.70
CSF	83.30	73.93	84.02	0.85	73.39	75.25	66.83	75.80	73.38	78.14
APOE + MMSE	79.37	93.25	72.97	0.89	68.29	81.73	76.68	70.23	81.24	78.05
Concatenation (all_features_set)	91.72	91.23	99.13	0.93	81.70	83.68	87.49	85.65	78.54	89.26

**Table 6:** 10-fold cross-validated classification performance for MCIs vs. MCIc.

Features measure	ELM				SVM-RBF			SVM-linear		
	ACC%	SEN%	SPE%	AUC	ACC%	SEN%	SPE%	ACC%	SEN%	SPE%
Cortical thickness	63.71	71.10	38.37	0.64	57.18	65.53	60.38	50.05	71.18	68.13
Surface area	61.43	72.28	74.80	0.69	54.25	63.22	76.17	58.27	72.23	64.85
Volume	67.85	84.40	87.73	0.74	59.17	78.28	67.55	62.14	74.45	57.73
CSF	71.37	78.84	68.57	0.72	64.73	60.09	72.78	67.33	78.82	68.78
APOE + MMSE	76.09	87.13	72.09	0.79	65.53	68.81	67.43	68.08	72.21	66.33
Concatenation (all_features_set)	83.38	93.01	75.77	0.85	71.37	81.16	76.75	75.71	78.38	84.67

**Table 7:** Comparison of classification of AD vs. HC and HC vs. MCI between the proposed method and existing state-of-the-art methods.

Author	Data	Classifier	Feature selection	AD vs. HC (%)	HC vs. MCI
Westman et al. [67]	MRI + CSF	OPLS	—	91.8	77.6%
Johnson et al. [68]	MRI + PET + CSF + cognitive scores	Stacked autoencoder	Sparse representation learning	95.9	85%
Hinrichs et al. [69]	MRI + PET + CSF + APOE + cognitive scores	MKL	—	92.4	n/a
Zhang and Shen et al. [39]	MRI + PET + CSF	SVM	Multitask feature selection	93.3	83.2%
Beheshti et al. [70]	sMRI	SVM	Feature ranking + genetic algorithm	93.01	—
Spasov et al. [71]	sMRI + cognitive measures + APOE + demographic	CNN	—	99.5	—
Maqsood et al. [72]	sMRI	CNN	—	92.85	—
Proposed method	MRI + CSF + APOE + MMSE	ELM	Filter (MRMR) + wrapper (SFS)	97.31	91.72%

**Table 8:** Comparison of classification of MCIs vs. MCIc between the proposed method and existing state-of-the-art methods.

Author	Data	Classifier	Feature selection	MCIs vs. MCIc (%)
Westman et al. [67]	MRI + CSF	OPLS	—	68.5
Zhang and Shen et al. [39]	MRI + PET + CSF	SVM	Multitask feature selection	73.90
Beheshti et al. [70]	sMRI	SVM	Feature ranking + genetic algorithm	75.00
Spasov et al. [71]	sMRI + cognitive measures + APOE + demographic	CNN	—	86
Moradi et al. [73]	MRI + age and cognitive		—	82
Proposed method	MRI + CSF + APOE + MMSE	ELM	Filter (MRMR) + wrapper (SFS)	83.38

## Data Availability

The Alzheimer's Disease Neuroimaging Initiative (ADNI) dataset (http://adni.loni.usc.edu) was used in this study. Complete information regarding ADNI investigators can be found at http://adni.loni.usc.edu/wp_content/uploads/how_to_apply/ADNI_Acknowledgement_ist.pd.
